# Effect of Gingival Hemostatic Agents on the Surface Detail Reproduction and Dimension Stability of Three Elastomeric Impression Materials

**DOI:** 10.1155/2023/6660721

**Published:** 2023-07-29

**Authors:** Sirichan Chiaraputt, Nattida Chokwattanapornchai, Katanyoo Limchaikul, Vibul Paisarnkobrit, Tool Sriamporn

**Affiliations:** ^1^Department of Conservative Dentistry and Prosthodontics, Faculty of Dentistry, Srinakharinwirot University, Bangkok, Thailand; ^2^Department of Prosthodontics, College of Dental Medicine, Rangsit University, Prathum Thani, Thailand

## Abstract

This study aimed to evaluate the effects of aluminum chloride based hemostatic agents on the surface detail reproduction and dimension stability. Three impression materials were investigated after contaminated with three commercial astringents with different concentration of aluminum chloride. The specimens from three impression materials were fabricated with a stainless-steel mold that followed the American Dental Association specification no.19. The mold was preliminarily contaminated with three hemostatic agents racestyptine, Dryz, and Expasyl™—and 80 specimens from each impression material—polyvinylsiloxane (PVS), polyether, and polyvinylsiloxane ether (PVSE), were fabricated and subjected to each astringent. The surface detail reproduction was examined using a stereomicroscope at 4x magnification, and the dimensional stability was analyzed at 24 hr with a measuring microscope. The surface detail data were statistically analyzed with Fisher's test at a significance level of 0.05. The dimensional stability data were analyzed using two-way ANOVA and Tukey's test at a significance level of 0.05. Aluminum chloride hemostatic agents can affect the surface detail reproduction of impression materials differently (*p* < 0.05). Thus, the first null hypothesis was rejected. PVS showed the highest percentage of satisfactory surface detail regardless of the hemostatic agent used in this study. PVSE showed a reduced percentage of satisfactory surface detail when the concentration of aluminum chloride was high. The three hemostatic agents affected the dimensional stability of each material differently (*p* < 0.05). Therefore, the second null hypothesis was also rejected.

## 1. Introduction

Indirect restorations, such as crowns, bridges, inlays, and onlays, require extraoral steps. The impression technique provides imprints for working models and other steps in the laboratories. The margin of tooth preparation is one of the key factors for a successful laboratory process. The impression plays an important role in providing a well margin-defined working model. A good impression technique should provide an imprint with high-quality surface detail reproduction. Hemostatic agents have been used with retraction cords to provide a suitable environment for impression taking [1].

Hemostatic agents can be classified into vasoconstrictor and astringent types. The frequently used astringents are aluminum chloride and ferric sulfate [1]. Although the astringent group can successfully control the moisture around the margin, the materials have been reported to be highly acidic. The pH value of astringents ranges between 0.7 and 2.0 [2]. This low pH can produce acid resistant surface on dentin and interfere the bonding procedure [3].

Sulfur in latex gloves or rubber dams can inhibit the polymerization of polyvinylsiloxane (PVS) impression materials. Incomplete polymerization creates a sticky surface and incomplete texture, affecting the model casting process [4]. Some hemostatic agents also contain sulfur, such as aluminum sulfate and ferric sulfate. Concerns have been raised that these hemostatic agents might affect all elastomeric impression materials. Hence, aluminum chloride containing astringent was recommended to avoid the effect of sulfur.

Studies have compared the effects of hemostatic agents on the setting reaction of PVS impression materials. Surface detail reproduction and dimension stability were evaluated. It was found that ferric sulfate and aluminum chloride decreased the surface detail reproduction. However, the dimension stabilities were in an acceptable range with a contraction rate of less than 0.5% [5–7]. In contrast, some studies have reported that hemostatic agents did not affect the polymerization and dimensional stability of impression materials [8–10]. However, there are no conclusions on how aluminum chloride affects the polymerization of impression materials. Previous studies have compared the effects of aluminum chloride and other hemostatic agents. There have been no studies of the concentrations of aluminum chloride in hemostatic agents, which could affect the polymerization of elastomeric impression materials. Additionally, studies of the effects of aluminum chloride hemostatic agents on polyether and polyvinylsiloxane ether (PVSE) remain limited. This study, therefore, intended to investigate the effects of aluminum chloride hemostatic agents on the polymerization of elastomeric impression materials. An in vitro study was performed to evaluate the surface detail reproduction and the dimensional stability of polyether, PVS, and PVSE impression materials. The null hypothesis of the study was that aluminum chloride hemostatic agents did not affect the surface detail reproduction and dimensional stability of materials.

## 2. Materials and Methods

**Figure 1:**
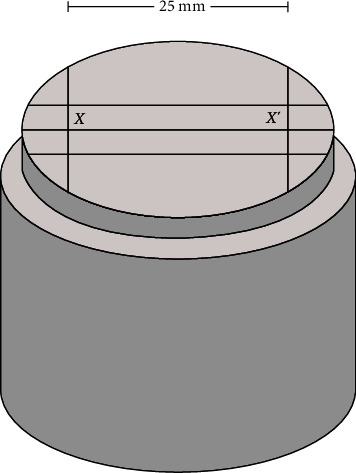
Diagram of ruled block dimension.

Stainless steel mold was created according to American Dental Association specification no.19 (ADA no.19,1977) for elastomeric impression materials [11]. The mold included ruled blocks and impression material molds. A ruled block consists of three horizontal lines and two vertical lines. All of these lines were expected to appear on the imprint of impression material (Figure 1).

**Figure 2:**
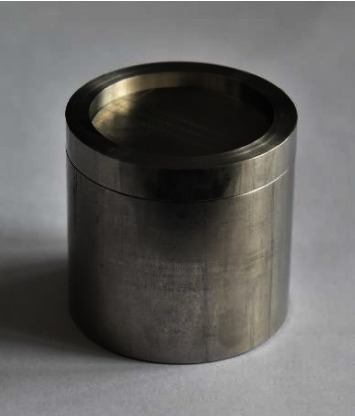
Stainless steel mold with ruled block.

The stainless-steel mold (Figure 2) was cleaned with alcohol, rinsed with water in an ultrasonic cleansing machine, and air dried before use. Hemostatic agents were applied according to the manufacturer's instructions into the mold according to Table 1. The applied hemostatic agent was left to contact the lines on the mold for 3 min. Then, the mold surface was rinsed for 60 s and air dried. Impression materials were mixed with an auto-mixed cartridge and applied with mixing tips to sample blocks until they were filled. Then, the tested blocks were covered with polyethylene sheets and 400 g metal sheets on top. The impression materials were left until complete setting (Table 2) with 3 min additional time according to ADA no.19.

The tested groups were divided according to impression materials. The groups were as follows: PVS (Silagum-Light), polyether (Impregum™ Garant™ L Duosoft) (PE) and PVSE (Identium® Light). About 80 impressions from each material were taken. The 20 specimens of each material group were contaminated with three hemostatic agents, and 20 specimens were set without contamination. The hemostatic agents were racestyptine, Dryz®, and Expasyl™.

The surface detail reproduction and dimension stability of the specimens were evaluated. The surface detail reproduction was evaluated under a stereomicroscope (Stereomicroscope, CX31, Olympus, Tokyo, Japan) with 4x magnification immediately after sample preparation. The evaluation criteria were satisfactory and unsatisfactory. Specimens with two of three horizontal lines along 25 mm with clear and continuous imprint were classified as satisfactory. The intrareader reliability was analyzed with Kappa correlation analysis and confirmed through a random pilot test on 10 impressions.

Fisher's exact test was performed with a significance level of *α* = 0.05. The assessment of dimension stability of the specimens involved analyzing the differences in these values. The initial distance between *X* and *X*′ (as depicted in Figure 1) was denoted as *A* and measured immediately following the intervention. Subsequently, the distance between *X* and *X*′ after a 24 hr period was labeled as *B*. Three measurements were conducted for each specimen, and the average value was recorded. The measurements were performed using a measuring microscope (MM-11, Nikon, Tokyo, Japan) at a magnification of 10x. The precision of the measurement was up to the nearest 0.001 mm, and this procedure was repeated three times to ensure accuracy. The dimensional change was calculated by

(1) $$\begin{array}{c} \text{Dimensional change}\left(\%\right)=\left(\left(A-B\right)/A\right)\times 100. \end{array}$$

Data were analyzed by two-way analysis of variance and Tukey's test at a significance level of *α* = 0.05.

## 3. Results

**Figure 3:**
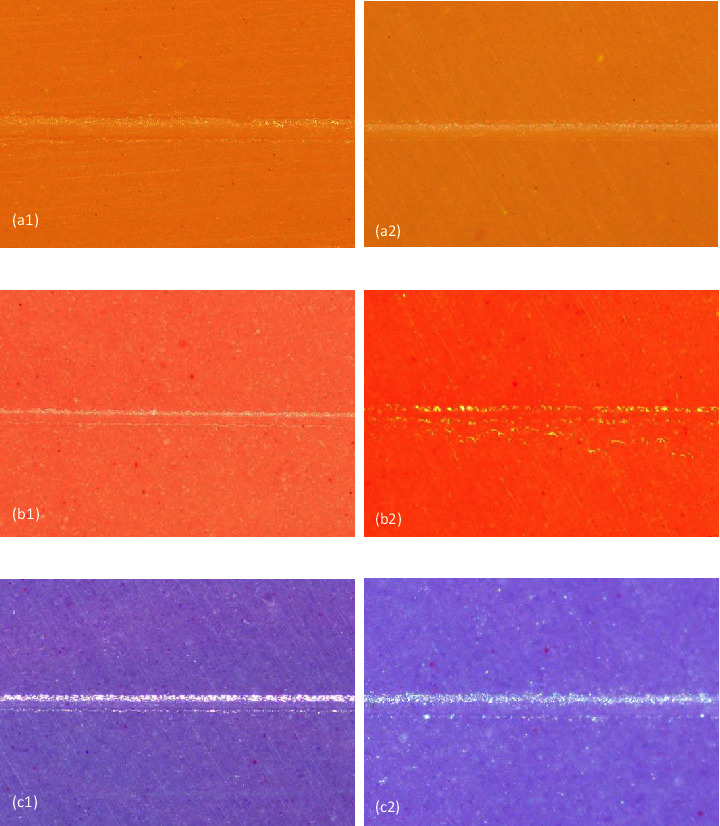
Surface details reproduction of elastomeric impression materials: (a) PVS, (b) PVE, and (c) PVSE. (a1, b1, c1) satisfactory (specimens with two or three clear and continuous lines along 25 mm); (a2, b2, c2) unsatisfactory (specimens excluded from satisfactory criteria).

**Figure 4:**
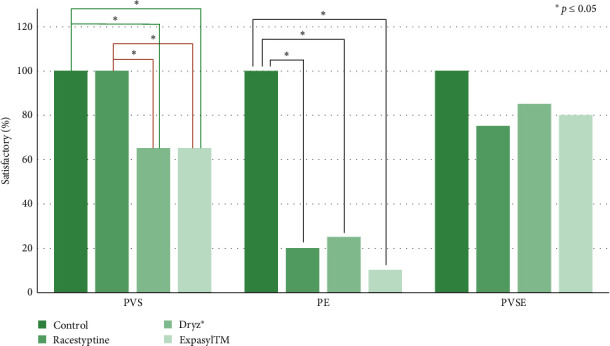
The statistic result of tested group at *p* ≤ 0.05 ( ^*∗*^*p* ≤ 0.05).

Surface detail reproduction data according to ADA no.19 are shown in Figure 3. The descriptive results were then analyzed by Fisher's exact test to prove the hypothesis. The results are shown in Figure 4. The replication ability of PVS was significantly different from that of polyether (PE) when contaminated with aluminum chloride hemostatic agents (*p* < 0.05). PVS contaminated with DryZ and Epaxyl showed 65% satisfactory results, while the group contaminated with racestyptine and the controls showed 100% satisfactory results. PE contaminated with racestyptine, DryZ, and Expaxyl showed satisfactory surface detail reproduction percentages of 20%, 25%, and 10%, respectively. The result in the contaminated group was significantly different from that in the control group, which showed 100% satisfaction. PVSE showed a high-satisfactory percentage that was not significantly different from the control group. The satisfactory percentages were 75%, 85%, and 80% for racestyptine, DryZ, and Expaxyl, respectively.

**Figure 5:**
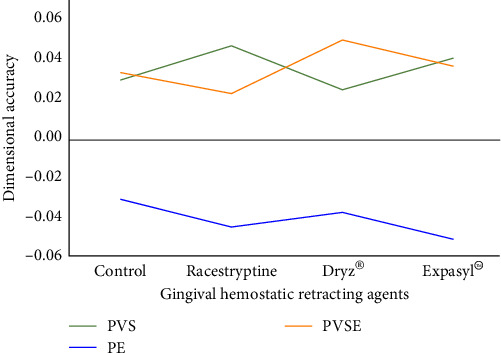
The mean dimensional stability of each impression material according the hemostatic agents.

The dimensional stability of impression materials is shown in Table 3 and Figure 5. Since there are two independent variables in this study therefore two-way ANOVA was initially used to analyze the data. The results indicated that none of the tested hemostatic agents significantly affected the whole sample groups in whereas the type of impression material affected the results. Tukey's test was then performed to indicate the differences among tested groups in the same material. Within the same impression material, the astringents affected the dimensional stability. Therefore, the null hypothesis was rejected. Nevertheless, the dimensional change in this study did not exceed 0.5%, which is within the ADA no.19 specification limit.

## 4. Discussion

The results from this study indicated that all noncontaminated groups and all racestyptine-contaminated groups provided significantly better surface detail than the DryZ and Expasyl groups. Therefore, the first null hypothesis was rejected. Racestyptine in solution showed a similar result in a previous study [10]. It was reported that Hemostop in solution form with aluminum chloride 25% did not affect the polymerization process of tested PVS impression materials. However, another study reported different results. Hemodent with 21.3% aluminum chloride and Gingi-Aid with 21.3% aluminum chloride showed poor surface detail reproduction in all PVS specimens [5, 7]. However, those studies did not clean the hemostatic agents according to the manufacturers' instructions. DryZ provided poor surface detail reproduction at 35%, which conformed to a previous study. In that study, PE showed significantly higher polymerization inhibition than PVS (Panasil and Express). Expasyl was reported to have a higher inhibition ability than other astringent materials. Moreover, Expasyl provided a higher polymerization rate after cleaning with hydrogen peroxide [12, 13].

PVS contaminated with DryZ and Expasyl exhibited significantly different polymerization from the racestyptine group. It should be noted that racestyptine is in solution, whereas Expasyl and DryZ are present in paste. DryZ retraction paste has cellulose gum in the component, which is the same binding agent as in toothpaste. Cellulose gum is a hydrophilic colloid that prevents the separation of solids and liquids in the paste [14]. Expasyl is composed of kaolin, which has a clay-like texture. The high viscosity in DryZ and Expasyl could cause difficulty in the material rinsing. Therefore, more remaining materials could be found compared with racestyptine.

It was found that PVS provided poor surface detail reproduction after contamination with DryZ or Expasyl and rinsing. When aluminum chloride is hydrolyzed, the agent is changed to hydrochloric acid [15]. Hydrochloric acid can break bonds between methyl groups (CH_3_) and silicon (Si). It was found that more methyl groups caused more bond cleavage [16], which could cause poor surface detail reproduction in PVS impression materials.

For polyether, the surface detail reproduction in these groups was significantly lower than that in the control group. The result conformed to previous studies [7, 10, 13]. The studies have reported polymerization inhibition, which results in poor surface detail reproduction. However, Vohra et al. [12] in 2020 reported only 20% surface detail reproduction from monopolyether impression material when contaminated with DryZ. The result could be affected by the product itself.

The effect of aluminum chloride on polyether can be described by the structure of polyether. Polyether is a copolymer of tetrahydrofuran and ethylene oxide in which the bond can be broken with a strong acid [17]. The strong acid can hydrolyze aluminum chloride and cause hydrochloric acid [18]. Hydrochloric acid has a high-dissolution rate, which may cause poor surface detail in polyether impression materials.

Information about the effect of hemostatic agents on PVSE is still limited. It was found that Alustin (20% aluminum chloride solution) increased the setting time of impression materials, whereas gingiva liquid (10% aluminum chloride solution), and racestyptine (25% aluminum chloride solution) affected the setting time very little [19]. However, hemostatic agents affected the surface detail production of PVSE with no significant difference in this study. However, the chemical structure of this impression material came from the combination of polyether and PVS [20]. The result found in this study is clearly different from polyether. A study reported that PVSE (Identium) provided less contact angle on the surface than PVS and polyether [17]. This result indicated the hydrophilic properties and flow of PVSE (Identium). Identium has a surface eraser surfactant and wetting conditioner surfactant, which may help the surface detail reproduction of the material.

Two-way analysis of variance indicated that hemostatic agents did not affect the dimensional stability of the tested impression materials. However, the type of impression material significantly affected the dimensional stability. Polyether provided a negative dimension stability value that was significantly different from that of PVS and PVSE. Polyether showed the greatest dimensional change percentage, whereas PVS showed the lowest dimensional change percentage. However, the dimensional change of all tested materials was in accordance with ADA no.19 which indicating a dimensional change of less than 0.5% within 24 hr [2]. The results in this study also showed dimensional changes within the International Organization for Standardization (ISO) 4823 standard [21]. ISO 4823 for elastomeric impression materials indicates a dimensional change of less than 1.5% within 24 hr. The results of this study conformed with those of previous studies [5–7].

This study found that PVS impression material and PVSE contracted in the same manner. The contraction of impression materials is caused by setting reactions that link and arrange internal bonds of the polymer structure [22]. Studies have reported the contraction of PVS contaminated with aluminum chloride, which conformed to the present study [6, 7]. Polyether showed expansion in this study, in contrast to a previous study [6]. Raipure and Kharsan [6] reported that polyether contracted after contamination with aluminum chloride. However, that study evaluated dimensional stability at 1 hr after setting, while the present study evaluated dimensional stability at 24 hr after setting. The differences in measurement time could cause different dimensional changes in materials. Generally, polyether absorbs water and causes expansion of the material [23]. Although the specimens in this study were stored in closed containers, polyether specimens can absorb water from the environment. A study reported that Impregum expanded even when impressions were taken in dry conditions at 24 hr after setting [24].

## 5. Conclusion

Aluminum chloride hemostatic agents significantly affected the surface detail reproduction of the three impression materials. Within the same impression material, the different concentration of aluminum chloride in astringent could produce significantly different dimensional stability except in polyether groups.

## Figures and Tables

**Table 1:** Hemostatic agents used in this study.

Gingival hemostatic retracting agents	Manufacturer	Type of materials	Composition	Batch #
Racestyptine (25% aluminum chloride)	Septodont, Saint-Maur-des-Fosses, France	Solution	Aluminum chloride hexahydrate, oxyquinol, hydroalcoholic	B25894AB
Dryz® (20% aluminum chloride)	Parkell, Edgewood, USA	Paste	Aluminum chloride hexahydrate, filler, cellulose gum	19176
Expasyl™ (15% aluminum chloride)	Acteon, Bordeaux, France	Paste	Aluminum chloride hexahydrate, kaolin, patent blue V, excipients	8874

**Table 2:** Working time and setting time of impression materials.

Product	Manufacturer	Type of material	Batch #	Working time (min)	Intraoral setting time (min)
Silagum-Light	DMG, Hamburg, Germany	Polyvinylsiloxane	226580	≤ 2.15	≥ 3.30
Impregum™ Garant™ L Duosoft™	3M ESPE, MN, USA	Polyether	7478882	2.00	3.30
Identium® Light	Kettenbach dental, Kettenbach, Germany	Polyvinylsiloxanether	200281	2.00	3.30

**Table 3:** Mean and standard deviation of the dimensional stability of impression materials.

Elastomeric impression materials	Gingival hemostatic retracting agents			
	Control	Racestyptine	Dryz®	Expasyl™
PVS	0.0302 ± 0.0202^A,B^	0.0475 ± 0.0253^A^	0.0253 ± 0.0159^B^	0.0413 ± 0.0217^A,B^
PE	−0.0299 ± 0.0239^E^	−0.0439 ± 0.0258^E^	−0.0365 ± 0.0335^E^	−0.0501 ± 0.0301^E^
PVSE	0.0340 ± 0.0254^C,D^	0.0234 ± 0.0178^C^	0.0504 ± 0.0309^D^	0.0372 ± 0.0332^C,D^

Capital letters indicated the significantly different dimensional stability of same elastomeric impression material after Tukey's test at *p* ≤ 0.05. The same capital indicated the same significant level.

## Data Availability

The data collected by researchers has been statistical analyzed and presented in the text. The raw data supporting the findings of this study will also be available from the corresponding author on request.
